# A Glimpse of the Sports Nutrition Awareness in Spanish Basketball Players

**DOI:** 10.3390/nu14010027

**Published:** 2021-12-22

**Authors:** Ignacio Escribano-Ott, Juan Mielgo-Ayuso, Julio Calleja-González

**Affiliations:** 1 Department of Physical Education and Sport, Faculty of Education and Sport, University of the Basque Country, 01007 Vitoria, Spain; julio.calleja.gonzalez@gmail.com; 2 Department of Health Sciences, Faculty of Health Sciences, University of Burgos, 09001 Burgos, Spain; jfmielgo@ubu.es

**Keywords:** recovery, sports performance, health, nutrition, basketball, sports nutrition, nutritional knowledge, nutritional behavior, nutritional education

## Abstract

Basketball is a team sport, with many fans and practitioners worldwide from all ages and levels. In all cases, players accumulate high levels of fatigue, and there is also limited time to recover between games or practices. In particular, nutrition plays a key role in optimizing performance and recovery. However, it is typical to observe erroneous nutritional behaviors among basketball players. It has been theorized that these behaviors are influenced by habits acquired based on the individual’s knowledge. Therefore, the main aim of this study was to conduct a descriptive research of the sports nutrition knowledge and practices in a sample of Spanish basketball players, from athletes under 18 years old (*n* = 69) to nonprofessional (*n* = 14) and professional adult players (*n* = 21). The sample was comprised of 49 men and 55 women. This was a transversal, cross-sectional, observational and descriptive study. All participants (*n* = 104) completed an anonymous online survey in order to analyze their sports nutrition knowledge and practices. In view of the obtained results, we can conclude that the knowledge of sport-specific nutrition in players under 18 years old, as well as non-professional and professional adult basketball players, is insufficient through all the categories and levels. The lack of professional support and time management difficulties were identified as some of the main barriers.

## 1. Introduction

Basketball is a team sport with many fans and practitioners worldwide [1], whose media impact can be partially explained by the impressive expansion and globalization phenomena experienced over the last 30 years [2]. It is a dynamic and constantly evolving game that has undergone numerous modifications since James Naismith created it in 1891 [3]. The changes introduced during the last decade significantly impacted the game, morphing it into a faster and more spectacular sport [4], and increasing the physical, psychological and physiological demands on the players [5,6]. Furthermore, the number of games played in professional basketball has increased considerably [7,8]. Given that, players can accumulate up to 70 games in approximately 34 weeks (FIBA), which implies up to 3 games per week with less than 72 h of recovery among them [9,10].

As a result of these changes, sports performance scientists have highlighted the need for a deeper understanding of the game [11] and the main demands experienced by the players [12]. Although most existing knowledge refers to men’s basketball [13,14], there is also an emerging trend to explore the singularity of female players (hormonal, biological and anatomical factors) due to the increased presence of women’s basketball in all categories and play level [15]. In this sense, more studies are needed, especially in the area of training [15]. Regarding youth players, the youngest athletes also face numerous challenges [16,17]. From the standpoint of nutrition science, younger athletes, especially those participating in high-performance training programs, must cope with physiological maturation needs and their athletic development [18]. For this reason, the special demands of the adolescent population have also attracted the interest of the scientific community [19,20].

In all cases, high levels of fatigue and the limited time to recover between games [11,12] have pushed teams to incorporate strategies to accelerate and optimize the recovery processes in their plans [21]. In fact, this challenge involves strategies representing a decisive competitive advantage [9].

Among all these recovery factors, nutrition plays a key role [22,23,24], covering the demands of the game, maintaining physical and psychological performance, and optimizing the athlete’s potential [25]. In particular, there is widespread knowledge that carbohydrates and proteins must be provided with specific timing, quantity and rhythm of administration [26] to enhance recovery [9]. Although it is difficult to establish a general nutritional guide that could suit all players, in a previous review [4], we suggested a theoretical-practical one, covering the key points that should be considered to optimize the player’s recovery through appropriate diet [22]. Despite the availability and accessibility of this theoretical knowledge to the majority of teams and their coaches, it is common to observe (in basketball and other team sports) that players still display erroneous behaviors, attitudes and beliefs [27,28]. Ultimately, this leads them to make undesirable food choices that can directly interfere with their performance and health [29]. It has been theorized that this behavior is influenced by the habits acquired based on personal knowledge [30]. In addition, age seems to be a direct factor influencing this choice behavior [31]. Although the scientific literature has previously investigated athletes and coaches’ nutritional knowledge [24,32,33], to the best of the authors’ knowledge, we did not find any previous studies conducted specifically for basketball, neither longitudinally nor transversally. We also did not find any previous studies conducted among different ages and categories, from young to professional players. Therefore, the main aim of this research was to conduct a descriptive study of the sports nutrition knowledge, practices and perceived barriers, in Spanish basketball players, from players under 18 years old (U-18) to non-professional (Non-pro) and professional (Pro) adult players.

## 2. Materials and Methods

This research was conducted from January to April 2021. All participants (Table 1) completed an anonymous online questionnaire sent by their coaches, managers or other players, guaranteeing their confidentiality.

### 2.1. Participants

The study sample consisted of 49 men and 55 women of U-18 categories who were 15–16 years of age (cadet-stage subgroup, *n* = 32) and 17–18 years of age (junior-stage subgroup, *n* = 37) and adult (17; 18). The sample was comprised of 49 men and 55 women within the adult category, of which 21 played in the highest Spanish competitions (Pro category), 10 in women’s leagues (LF1, LF2) and 11 in men’s leagues (ACB First League, LEB-2 Silver League). The remaining senior participants (Non-Pro category) belonged to teams playing in other federative divisions in Spain, with 8 women in the 1st national feminine division (3rd Division) and 6 men in the Liga EBA (4th Division). The inclusion criteria were: (1) players aged between 15 and 18 years with a Spanish federation license, or if they were older than 18 years old, to compete in Pro or Non-Pro category (FEB); (2) participation in at least 75% of the annual practice sessions; (3) regular participation in these competitions; (4) no recent injury (1 month before the beginning of the study); (5) absence of drugs at the time of the study. The following exclusion criteria were established: (1) lack of fluency in the language in which the questionnaire was delivered (Spanish).

### 2.2. Ethical Issues

The ethical approval was obtained from those responsible of the clubs and federations which participated in the study. They were informed of the purpose of the study and its potential benefits, its methodological nature and the used methodology. This study was approved by the Ethics Committee for Human Research (CEISH) of the University of the Basque Country (UPV/EHU) with code M10_2020_259 and conducted under the ethical principles of the Helsinki Declaration (actualization Fortaleza, 2013) [34].

### 2.3. Study Design

This is a descriptive, cross-sectional and observational study. An ad-hoc questionnaire was designed to collect the following information: category of play, sex, sports nutrition knowledge and sports nutrition practices. It consisted of a combination of question using checkboxes and open-ended responses. Before starting the study, the authors designed a preliminary draft. In addition, a pilot test was performed by two professional basketball team (one men’s basketball team and one women’s basketball team) who volunteered participate, which was not included in the study.

### 2.4. Data Collection Method

The survey sections consisted of these specific questions:

- Sports nutrition questionnaire: The questionnaire was previously validated and is considered a simple yet applicable tool to administer [35]. It contains 23 questions grouped into 6 different sections: (I) nutrients: questions (7 items) about the degree of agreement on experts’ recommendations and the profile of certain foods according to their predominant content of a nutrient; (II) hydration: questions (5 items) on rehydration strategies and the profile of a sports drink; (III) recovery: questions (5 items) on which foods may represent a suitable choice in terms of quantity and presence of certain nutrients, as well as the timing of intake; body mass management: questions on (IV) strategies for healthy weight gain (2 items) and (V) weight loss (2 items); (VI) supplementation: questions (2 items) about the degree of agreement on experts’ recommendations on supplementation. Each categorical and total knowledge characteristic was assessed by transforming the results obtained into a scale equivalent to the school grading system in Spain (https://www.boe.es/eli/es/o/2007/06/19/eci1845. Last accessed: 21 November 2021), where 0 was the minimum value and 10 the maximum value (Table 1).
- Sports nutrition practices: A questionnaire was configured to assess the sports nutrition practices by adapting the existing questionnaires of Heikura et al. [36] and Altarriba-Bartes et al. [27]. It contained a total of 36 items. Depending on the answer (yes or no), the questionnaire continued to the following question or jumped to the next section, so the total number of questions to be answered could be reduced. Items were grouped into the following thematic blocks: training adjustment according to competition objectives (7 items), pre- and post-training nutritional strategies (14 items), and pre- and post-match nutritional strategies (15 items). Each of these blocks contained questions about the main reasons for adhering or not to the strategies and the influence of other people on their behaviors to follow the recommendations [4]. The results are presented as arbitrary units (AU) following the grade marks system of the Spanish Education Ministry (https://www.boe.es/eli/es/o/2007/06/19/eci1845. Last accessed: 21 November 2021).

### 2.5. Statistical Analysis

The data were exported from the online survey (Google Forms^®^) to a spreadsheet (Microsoft^®^ Excel for Windows 10). The results are presented as means, standard deviation (SD) and frequencies. Normal distribution was tested using the Kolmogorov–Smirnov test (*n* = 104). In addition, the Levene test was used to analyze the previous homoscedasticity of the data. The comparison of means among independent samples was performed with the ANOVA test, and Bonferroni post-hoc analyses were conducted to identify the differences among groups. Categorical variables were analyzed using the Chi-square test, and these analyses were stratified by level of competition. The effect size, from Cohen’s D, was considered small when d = 0.20, medium when d = 0.50, and large when d = 0.80 [37]. Logistic regression analyses were used to determine the association among category-knowledge-behaviors. The magnitude of the correlation coefficients was determined as trivial (r < 0.1), small (0.1 < r < 0.3), moderate (0.3 < r < 0.5), high (0.5 < r < 0.7), very high (0.7 < r < 0.9), nearly perfect (r > 0.9) or perfect (r = 1) [37]. In all statistical tests, the statistical significance level *p* < 0.05 was used for bilateral contracts. Data analysis was performed with the statistical package SPSS^®^ for Windows version 26.0, SPSS INC, Chicago, IL, USA) and in the R (1.4 version) programming environment.

## 3. Results

### 3.1. Nutritional Knowledge

**Figure 1:**
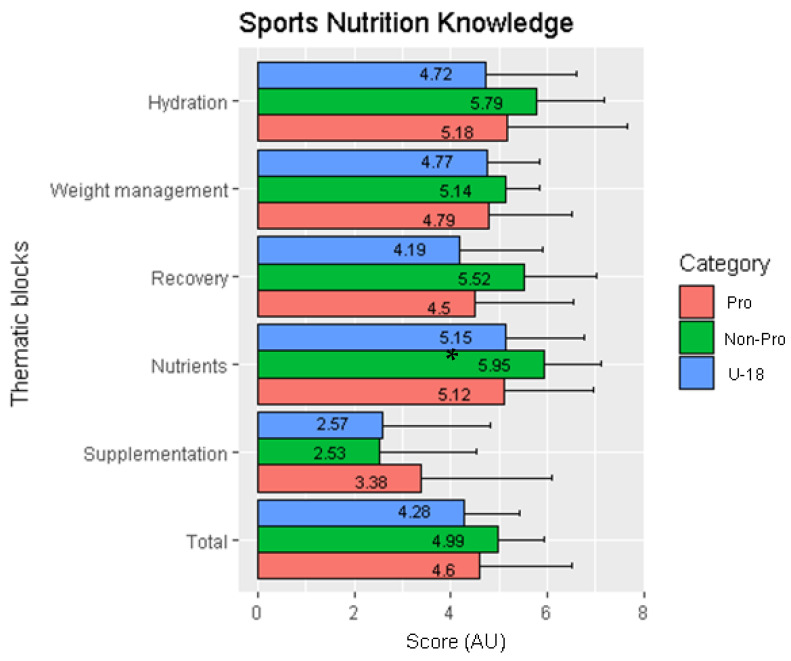
Sports nutrition knowledge (thematic blocks). Legend: * statistical significance difference.

**Figure 2:**
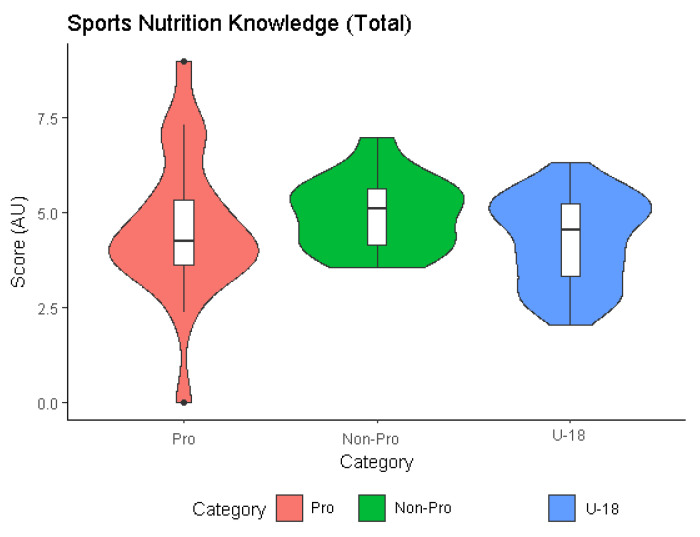
Sports nutrition knowledge (total).

A total of 104 participants completed a sports nutrition knowledge questionnaire (Table 2). The following thematic blocks (Figure 1) were analyzed: hydration (U-18: 4.72 AU vs. Non-pro: 5.79 AU vs. Pro: 5.18 AU: ns), recovery (U-18: 4.19 AU vs. Non-Pro: 5.52 AU vs. Pro: 4.5 AU: ns), body mass management (U-18: 4.77AU vs. Non-Pro: 5.14 AU vs. Pro: 4.79 AU: ns) and supplementation (U-18: 2.57 AU vs. Non-Pro: 2.53 AU vs. Pro: 3.38 AU: ns) (Figure 2). With respect to the nutrient (U-18: 5.15 AU vs. Non-Pro: 5.95 AU vs. Pro: 5.12 AU: ns), significant differences in logistic regression were found between categories (Non-Pro vs. Pro vs. U-18: *p* = 0.03) with a high effect size (d = 0.82).

### 3.2. Nutritional Practices and Strategies

**Figure 3:**
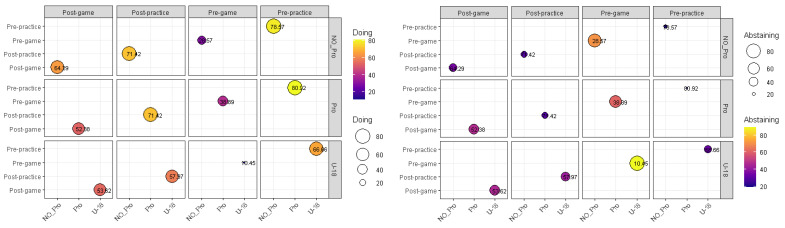
Sports nutrition practices. This figure compares the percentage of players following or not following the nutritional practices.

To analyze the nutritional practices and behaviors (Table 3), all the participants completed the same adapted questionnaire used in previous work. The results (Figure 3) show that 83% of participants did not adjust their nutrition for the work required (U-18: 81.16% vs. Non-Pro: 78.57% vs. Pro: 76.19%: ns). However, 71.15% of participants (U-18: 66.66% vs. Non-Pro: 21.42% vs. Pro: 19.04: ns) reported paying attention to their pre-training nutrition, and 62.5% (U-18: 57.97% vs. Non-Pro: 71.42% vs. Pro: 71.42%: ns) to post-training nutritional recovery. These frequencies decreased when they were asked about their match-day practices, where 18.18% of participants reported paying attention to pre-competition nutrition (U-18: 10.45% vs. Non-Pro: 28.57% vs. Pro: 38.89%: ns) in contrast to 54.81% (U-18: 46.38% vs. Non-Pro: 35.71% vs. Pro: 47.62%: ns) of those who did so at the end of the match.

### 3.3. Perceived Barriers

Although the correlation magnitude was small (r = 0.241), we found a positive association between knowledge levels and adherence to the practices recommended by the experts (*p* = 0.035; r = 0.207; small), and a better fit to these as the categories became more professional (*p* = 0.14; r = 0.241; small).

## 4. Discussion

This descriptive research aimed to describe the nutritional knowledge, behaviors, practices and perceived barriers of basketball players of different categories and levels. In view of the results obtained in this study, we can conclude that sports nutrition knowledge is insufficient among basketball players of all levels and ages, resulting in inadequate eating practices. In addition, there are barriers, such as lack of professional support and time management difficulties, that make it difficult for players to optimize their nutrition.

### 4.1. Nutritional Knowledge and Practical Management of Nutrients

Sports nutrition is a rapidly growing and evolving science [38]. There is a strong agreement that nutrients play a key role in the acute, adaptive and chronic response to exercise, and the availability and functioning of energy systems [22,38]. Moreover, the type of nutrient, knowledge and management of the timing and amount of intake is critical [39]. Likewise, sports nutrition must respond to the specific mechanisms that generate fatigue [4] and the fatigue profiles generated by each playing position, particularly in team sports [40]. In this sense, our study found no significant differences in the theoretical knowledge of nutrients among categories, obtaining an average score for the 3 groups of 5.25 out of 10 maximum total points. To the best of the authors’ knowledge, this is the first study that specifically addresses this variable in basketball while including professional players. These scores follow the direction of work conducted in other sports disciplines, where other athletes also scored similarly [24,41,42,43]. In addition to describing knowledge, our description also addressed the practical handling of knowledge by the athletes. Adjusting the availability and supply of nutrients, especially carbohydrates, to the daily demands is an aspect that has generated great interest inside the specialized scientific community [39,44,45], highlighting the need not to adopt nutritional practices exclusively oriented toward competition [46]. Our study found significant differences between those who did adjust their diet according to training, with the group of athletes who did not do so being larger (YES: 20.19% vs. NO: 79.81%). This fact highlights that, despite the brief time since theoretical knowledge on nutritional periodization has emerged [47,48,49], only in very specific cases, such as those recorded by Heikura in 2018 [36], is it being transferred to practice. In this sense, future research lines could explore which characteristics are fundamental to adjust nutritional periodization [46] to each sporting context or the main barriers that limit the transfer of this theoretical knowledge to field practice, particularly in basketball and team sports.

### 4.2. Nutritional Knowledge and Practical Management of Supplementation Strategies

Sports supplements are becoming increasingly popular among athletes, with a broad and accessible range of products available [23]. The research conducted by our research group found that some sports supplements could be of interest for health [50], while others, due to their proven ergogenic effect, could be beneficial for sports performance [51,52,53]. The availability of complex supplementation makes it necessary for the basketball players to tailor their supplementation menus individually [54], selecting those that are genuinely beneficial for their practice [4], and considering their own individual parameters based on their feels and preferences [55]. Particularly important are those related to the sex variable, since most of the published studies have been performed among men [51], and exhaustive research is needed on the female population that considers their physiological peculiarities, mainly hormonal [52]. To accurately apply these strategies, it is necessary for players to have sufficient knowledge that will allow them to optimize their performance and protect their health and enlarge their sporting careers [56]. In that way, our research has shown that basketball players’ knowledge about sports supplementation does not reach the “pass” category, registering a value of 2.73 out of a maximum value of 10. These records were repeated in the three analyzed categories (U-18: 2.57 vs. Non-Pro: 2.53 vs. Pro: 2.74). In particular, in the case of the U-18 category, these scores could be justified because, initially, this group should not require their use, as a varied, balanced diet that adheres to a Mediterranean style could theoretically be sufficient [57,58]. However, Chiba et al. [59] found that as young people progress in their development, their consumption of nutritional supplements also increases. Most of the time, supplements are incorrectly used, so early educational interventions [59] could be a measure of interest. Regarding the senior population (Non-Pro and Pro), our results are in line with other similar studies that have found that athletes present have a low level of knowledge [25,60] or that they are unaware of the need for an independent laboratory to guarantee the purity and safety of a product [61,62]. This lack of know-how demonstrates how exposed athletes are to brands’ promises of product improvements [54], and how they cannot protect themselves from these attractive messages by asking basic questions about the safety, efficacy or risks of using them [63].

### 4.3. Nutritional Knowledge and Practical Management of Hydration Strategies

One factor that directly affects the onset of fatigue is dehydration [4,64], so it is essential to achieve a homeostatic balance between fluid losses and exogenous fluid replacement [64,65]. Hydration plays a vital role in reducing explosive performance during basketball competition [66] and should be a key focus on recovery strategies. On the other hand, very high or sustained losses over time, in addition to negatively affecting athletic performance, can put health at serious risk. Therefore, a widely accepted rehydration strategy is to ingest an amount of fluid equivalent to twice the weight lost [22,23,24,25,26,27,28,29,30,31,32,33,34,35,36,37,38,39,40,41,42,43,44,45,46,47,48,49,50,51,52,53,54,55,56,57,58,59,60,61,62,63,64,65]. However, adult players showed better knowledge about hydration management (U18: 4.72 AU vs. Non-Pro: 5.79 AU vs. Pro: 5.18 AU). Despite that finding, we did not find significant differences when comparing their practices to U-18’s. This lack of theoretical knowledge could also be reflected in subsequent hydration behavior, as has been described in other studies [66,67]. It is difficult to establish a relationship that could explain these differences. It may be theorized that older players could have received a greater number of messages about the importance of following adequate hydration. In the last two decades, hydration has been a subject widely addressed by the scientific community [68]. Consequently, transferring and communicating these recommendations into practice is relatively easy for coaches [69].

### 4.4. Nutritional Knowledge and Practical Handling for Body Mass Management

Basketball players’ bodies are large and strong regardless of their play position [70,71,72,73,74], so their diet must provide enough nutrients and energy to maintain adequate levels of fat and muscle [73]. Our study found no significant differences by category, recording a mean value of 4.83 AU (U-18: 4.77 AU vs. Non-Pro: 5.14 vs. Pro: 4.79) with 10 being the maximum possible. This lack of knowledge about the management of body composition could be related to an inadequate diet that does not comply, either by excess or deficiency, with the experts’ recommendations [75,76]. In the case of female basketball players, there is a profound need to understand precisely which nutritional strategies are the most appropriate to achieve optimal body composition. Their specific hormonal profile, as well as the fluctuations that occurs during the menstrual cycle [77], mean that physiological complications derived from a prolonged state of energy efficiency can place their health at risk [78].

### 4.5. Nutritional Knowledge and Practical Handling of Recovery Strategies

The organic and psychological recovery of the athlete is a task that requires high precision and individualization [21], and depends directly on the complex, interconnected and specific dimensions of each sport [9,79,80,81]. Among these tasks, nutrition plays a determining role in accelerating and optimizing recovery [82,83]. It is one of the main methods used by teams [82], although this knowledge needs to be further expanded to design specific strategies for the female population [84]. Our study found significant differences in knowledge related to nutritional recovery strategies between younger and adult players (*p* = 0.03). This may be due to a better perception by the players themselves or their teams of the need to recover more quickly and efficiently [4,5], and thus cope with the demands of training and matches. Alternatively, this knowledge may be attributed to structures where nutritional recovery practices are imposed by the sporting structure itself [27]. In contrast, Bird and Rushton [85] observed in a recent study that the mean value of this knowledge did not reach the cut-off of 5 out of 10 players (U-18: 4.19 AU vs. Non-Pro: 5.52 vs. Pro: 4.5 AU). Therefore, in addition to facilitating and promoting practical access to nutritional recovery methods [4], these could be accompanied by training strategies that would give them greater autonomy, positively impacting their performance and lives [55]. In addition to describing their knowledge, we also analyzed their nutritional practices in training and matches in our study. These practices have a direct impact on recovery, completing it and reaching a state of optimal preparation before exercise or starting the recovery process itself [86]. Although their knowledge reflected a lack of mastery of sport-specific recommendations, in their behavior, they reported paying attention to their nutrition before (YES: 71.15% vs. NO: 28.84%) and after (YES: 62.5% vs. NO: 37.5%) training. With this finding, it could be theorized that over and above the recommendations based on scientific evidence, athletes’ behavior is more influenced by their perception of how easy it is to carry out these strategies [87], or the sense of individual well-being that they generate [55].

### 4.6. Perceived Barriers to Nutritional Knowledge and Practices

Lack of professional support, difficulties in time management, and lack of knowledge are some of the main barriers perceived by athletes and coaches, which, in addition to compromising their performance, expose them to numerous agents that put their protection at risk [88]. These difficulties coincide with those found in our research, where the lack of knowledge offered by a professional was recorded as a common variable in all age categories for pre-match (U-18: 35% vs. Non-Pro: 40% vs. Pro: 36.36%) and post-match (U-18: 64.52% vs. Non-Pro: 40% vs. Pro: 44.44%) practices. On the other hand, time management depending on the residence situation was observed as a predominant barrier in the professional (Pro) category (PRE: 25% vs. POST: 33.33%). These results are in line with those obtained by Sekulic et al. (2019), where athletes also reported insufficient knowledge of recommended practices. Our results point in the same direction, as we found a positive association between the levels of knowledge and adherence to the practices recommended by the experts (*p* = 0.035), and a better adjustment to these as they scale the categories (*p* = 0.14). Thus, it seems essential that clubs and federations make the assistance of a professional nutritionist available to players. A professional nutritionist could help players and coordinate their personal circumstances [89] with those based on the nutritional demands of basketball [90].

## 5. Conclusions

In view of the obtained results, we can conclude that the sports nutrition knowledge and practices in this sample of Spanish Basketball Players, from U-18 to non-professional (Non-Pro) and professional (Pro) adult players, is insufficient and inadequate, which may compromise their performance and health. In addition, the lack of professional support, time management difficulties and lack of knowledge were identified as some of the main barriers that may prevent basketball players from developing healthy behaviors in line with expert’s recommendations.

## 6. Limitations, Strengths and Future Lines of Research

This study presents some limitations. First, the sample size is relatively limited in comparison with the absolute number of basketball licenses in the Spanish Basketball leagues. Nonetheless, this work represents a framework that can be expand in future research lines in a larger sample. Future research may also include the role of coaches, families and other social agents that could influence players’ nutritional knowledge and behaviors. In addition, future research may analyze specific age-groups or categories as Altarriba did [27], or could group players by gender.

The second limitation is that the questionnaires were completed by the players themselves. Future studies may include the questionnaire as a part of a nutritional intervention designed to improve this knowledge and players’ behaviors.

Finally, this study was conducted during a global pandemic.

## 7. Practical Application

Based on the findings, our research team has proposed some ideas that could help to build bridges between the theoretical knowledge and players’ practices.

### 7.1. Nutritional Education Strategies to Increase Knowledge

The dietary education of athletes is a key aspect that can promote appropriate eating behavior, with adolescence being an ideal time for its development. Although many possible interventions can be carried out, most of which are sufficient to achieve a positive impact, short interventions at specific times of the season (training camps, technical improvement, pre- and post-season) using new technologies (such as mobile applications) could be a strategy that could quickly increase the knowledge of nutrition. It could also be beneficial for coaches and technical staff to participate in this training due to the high impact that their behaviors and nutritional knowledge have on athletes.

### 7.2. Quick Applied Ideas to Boost Nutritional Practices

Facilitating practical solutions through popular forms of communication and making them visible in training areas or providing them directly to players could be an effective strategy to provide visual examples that can be quickly assimilated and implemented. These designs could show: (1) foods, preparations or menus that integrate the amount and type of nutrients needed for basketball practice, as well as when to take them; (2) hydration strategies through foods with a high hydration index, as well as ways to evaluate and measure their degree of dehydration and rehydration rate; (3) diagrams that help players decide and choose whether to follow a supplementation pattern as well as facilitating the ways to consult about the safety of a product; (4) specific infographics.

## Figures and Tables

**Table 1:** Description of the sample.

Category	Sub-Category	Men	Women
U-18	Cadet-Stage	17	25
	Junior-Stage	15	12
Non-Pro	EBA League	6	-
	1st National Feminine	-	8
Pro	LEB-2 Silver League	5	-
	ACB First League	6	-
	Feminine League 2	-	2
	Feminine League 1	-	8
TOTAL		49	55

Legend: U-18: player under 18 years old, Non-Pro: non-professional adult player, Pro: professional adult player, EBA: Liga Española de Baloncesto Aficionado (Spanish Amateur Basketball League), LEB: Liga Española de Baloncesto (Spanish Basketball League), ACB: Asociación Clubes profesionales de Baloncesto (Professional Basketball Clubs Asociation).

**Table 2:** Sports nutrition knowledge by category, gender and thematic blocks.

Category		*n*	Nutrients ^1^	Hydration ^1^	Recovery ^1^	Mass Management ^1^	Supplementation ^1^	Total ^1^
U-18	Men	32	5.3 (1.97)	5.07 (2)	4.66 (1.63)	4.83 (1)	2.79 (2.18)	4.53 (1,1)
	Women	37	5.01 (1.22)	4.41 (1.75)	3.79 (1.69)	4.72 (1.13)	2.38 (2.32)	4.06 (1.15)
	Total	69	5.15 (1.61)	4.72 (1.88)	4.19 * (1.71)	4.77 (1.06)	2.57 (2.25)	2.28 (0.54)
Non-Pro	Men	6	5.6 (1.54)	5.74 (1.48)	4.85 (1.24)	5.22 (0.65)	2.43 (1.79)	4.77 (0.93)
	Women	8	6.22 (0.82)	5.83 (1.43)	6.02 (1.53)	5.08 (0.79)	2.62 (2.25)	5.15 (1)
	Total	14	5.95 (1.17)	5.79 (1.39)	5.52 * (1.49)	5.14 (0.71)	2.53 (1.99)	2.43 (0.65)
Pro	Men	11	4.81 (2.11)	4.64 (2.57)	4.05 (2.2)	4.18 (1.93)	2.65 (2.49)	4.07 (2.04)
	Women	10	5.48 (1.53)	5.78 (2.33)	5 (1.83)	5.47 (1.25)	4.18 (2.88)	5.18 (1.68)
	Total	21	5.12 (1.84)	5.18 (2.47)	4.5 (2.04)	4.79 (1.73)	3.38 (2.73)	2.35 (0.81)
Total	Men	49	5.23 (1.94)	5.06 (2.07)	4.55 (1.72)	4.74 (1.26)	2.71 (2.17)	4.46 (1.34)
	Women	55	5.27 (1.29)	4.87 (1.91)	4.33 (1.87)	4.91 (1.13)	2.74 (2.47)	4.43 (1.32)
	Total	104	5.25 (1.62)	4.96 (1.98)	4.43 (1.79)	4.83 (1.19)	2.73 (2.32)	4.44 (1.32)
*p*-value	-	-	0.221	0.151	0.0386	0.567	0.361	0.151
d Cohen	-	-	-	-	0.82	-	-	-

Legend: SD: values are presented as mean (SD). Standard deviation; d, Cohen’s d (The effect size, from Cohen’s D, was considered small when d = 0.20, medium when d = 0.50, and large when d = 0.80); normal distribution ^1^; * statistically significant differences among groups (*p* < 0.05).

**Table 3:** Sports nutrition practices by category, gender and thematic blocks.

	Training ^1^ Adjustment *		Pre ^1^ Practice		Post ^1^ Practice		Pre ^1^ Game		Post ^1^ Game *	
	YES	NO	YES	NO	YES	NO	YES	NO	YES	NO
	fi (%)	fi (%)	fi (%)	fi (%)	fi (%)	fi (%)	fi (%)	fi (%)	fi (%)	fi (%)
U-18	13 (18.84)	56 (81.16)	46 (66.66)	23 (33.33)	40 (57.97)	29 (42.02)	7 (10.45)	60 (89.55)	37 (53.62)	32 (46.38)
Men	9 (28.13)	23 (71.88)	25 (78.12)	7 (21.85)	23 (71.87)	9 (28.12)	4 (13.33)	26 (86.67)	19 (59.38)	13 (40.63)
Women	4 (10.81)	33 (89.19)	21 (56.75)	16 (43.24)	17 (45.94)	20 (54.05)	3 (8.11)	34 (91.89)	18 (48.65)	19 (51.35)
Non-Pro	3 (21.43)	11 (78.57)	11 (78.57)	3 (21.42)	10 (71.42)	4 (28.57)	4 (28.57)	10 (71.43)	9 (64.29)	5 (35.71)
Men	1 (16.67)	5 (83.33)	5 (83.33)	1 (16.66)	5 (83.33)	1 (16.67)	1 (16.67)	5 (83.33)	3 (50)	3 (50)
Women	2 (25)	6 (75)	6 (75)	2 (25)	5 (62.5)	3 (37.5)	3 (37.5)	5 (62.5)	6 (75)	2 (25)
Pro	5 (23.81)	16 (76.19)	17 (80.92)	4 (19.04)	15 (71.42)	6 (28.57)	7 (38.89)	11 (61.11)	11 (52.38)	10 (47.62)
Men	3 (27.27)	8 (72.73)	9 (81.81)	2 (18.18)	8 (72.72)	3 (27.27)	4 (50)	4 (50)	8 (72.73)	3 (27.27)
Women	2 (20)	8 (80)	8 (80)	2 (20)	7 (70)	3 (30)	3 (30)	7 (70)	3 (30)	7 (70)
TOTAL	21 (20.19)	83 (79.81)	74 (71.15)	30 (28.84)	65 (62.5)	39 (37.5)	18 (18.18)	81 (81.82)	57 (54.81)	47 (45.19)
Men	13 (26.53)	36 (73.47)	39 (79.59)	10 (20.40)	36 (73.46)	13 (26.53)	9 (20.45)	35 (79.55)	30 (61.22)	19 (38.78)
Women	8 (14.55)	47 (85.45)	35 (63.63)	20 (36.36)	29 (52.72)	26 (47.27)	9 (16.36)	46 (83.64)	27 (49.09)	28 (50.91)
Chi square	0.877		0.362		0.408		0.362		0.817	

Legend: frequency (fi); normal distribution ^1^; * statistically significant differences (*p* < 0.05).

## Data Availability

The data presented in this study are available within the article.
